# Neurophysiology combined with sonography as a useful tool in median nerve leprosy diagnosis

**DOI:** 10.1055/s-0044-1801743

**Published:** 2025-01-08

**Authors:** Osvaldo J. M. Nascimento

**Affiliations:** 1 Universidade Federal Fluminense, Hospital Universitário Antonio Pedro, Departamento de Neurologia, Niterói RJ, Brazil.; 2 Academia Nacional de Medicina, Rio de Janeiro RJ, Brazil.

Carpal tunnel syndrome (CTS) is the most common compressive peripheral neuropathy worldwide, affecting individual quality of life and work-related activities, mainly in those living in low or middle-income countries. The incidence and prevalence of CTS vary in different target populations, with a higher rate in middle-aged individuals (50-59 years) and women, as recently corroborated in a study conducted in Taiwan with an approximate annual incidence of 0.4%. 1

In California workers, a total of 139,336 CTS cases were referred (incidence of 6.3 cases per 10,000 full-time workers), being in women (8.2) 3.3 times higher than that seen among men (2.5). 2 Specific data from Brazil is limited but reflects the role of occupational biomechanical overload in the development of CTS. 3 In Rio Grande do Sul State was observed a female-to-male ratio of 5.6:1 with a mean age of ∼48.3 years. Countryside population seems to be more susceptible due to agriculture and farming-intensive jobs. 4 Kouyoumdjian and Araujo (2006) evaluated CTS electrodiagnosis in 3.125 consecutive patients. 5 They found 43 cases (1.38%) associated with manual milking (mean age was 44.9 years). The most interesting finding was the male prevalence (88.4%); manual milking work is practically a male job; it reflects the role of occupational biomechanical overload in CTS. 5 Even though CTS pathophysiological mechanisms are not fully understood, the existence of an underlying subclinical neuropathy is a factor that may increase the susceptibility for the median nerve entrapment. Metabolic, hormonal disturbances, obesity, pregnancy, hereditary diseases, inflammation, and other associated conditions can be implicated.

Leprosy, a chronic infectious disease caused by *Mycobacterium leprae (M leprae),* is one of the most common treatable peripheral neuropathies in the world, being India and Brazil the most endemic countries. Leprosy is a neuritis of immunological origin that results in autonomic, sensory, and motor neuropathy. 6 It can be effectively treated, including multidrug therapy, before disability appears. The risk of irreparable deficiencies is associated with late diagnosis. *M. leprae* has a strong tropism for Schwann cells, consequently resulting in progressive axonal damage. 6 The neurological clinical presentations of leprosy have been related in 2013, 6 including six cases of the rare CTS presentation. 6 7 These cases of isolated median neuropathy were the first sign of leprosy, selected from patients with an exclusive median sensory complaint. Three of the patients had skin lesions, and three had pure neuritic leprosy (PNL). 8 Clinical median nerve function impairment was confirmed by neurophysiological testing and histopathology. We highlighted the importance of early diagnosis so that the patient can receive adequate clinical treatment that consequently can avoid permanent disability. 7

Recently, Naggapa et al. (2021) in a study with high-resolution sonography technique called attention to median nerve enlargement 2-cm proximal to the distal wrist crease in 26 patients, distinguishing leprosy from CTS. 9 They concluded that this important discriminating sign can be used at point-of-care to identify patients with leprosy.

In Brazil, leprosy cases are also more prevalent in low-income areas, mainly in the countryside and in the Amazon region, where CTS are frequent. It should be noted that the diagnostic instruments used, the electromyography and high-resolution ultrasound devices, are expensive and require highly specialized and trained professionals, who are difficult to find in the regions with the highest prevalence of these two conditions in developing countries.

In this issue of *Arquivos de Neuro-Psiquiatria* , Alves and colleagues offer a very important and practical contribution revisiting the median nerve electrodiagnostic and high-resolution sonography findings in patients with CTS or leprosy in the article entitled “Median nerve impairment in leprosy: how does it differ from the classic carpal tunnel syndrome?” 10 . The Brazilian contribution to leprosy knowledge has worldwide recognition. The importance of electrodiagnostic studies, recently associated with nerve imaging with high resolution sonography, brings a better understanding of the involvement of different nerve trunks that facilitates a more precise differential diagnosis and the best treatment window for leprosy. This is the focus of Alves and colleagues, considering the PNL modality. 10 There were 29 patients included (CTS: 14; PNL: 15), with a female predominance (55.2%; 16/29). 92.8% of patients with CTS and 80.0% with PNL had bilateral impairment of the median nerve, with a total of 27 nerves affected in each group. This study is the first to evaluate the morphological and functional impairment of the median nerve in a combined and systematic way, favoring a better etiological diagnosis of PNL. 10 They conclude that the presence of neural thickening and demyelinating involvement in the segments proximal to the carpal tunnel favors the diagnosis of leprosy. This methodology could be applied to other nerve trunks favoring the differential diagnosis of leprosy, including its challenging PNL form of presentation. Inflammatory chronic autoimmune neuropathies, other infectious neuropathies, inherited neuropathies, and others can be benefited with this dual methodology facing a clinical CTS case in endemic areas, as elegantly shown by Alves et al. 10
